# High-titer modular retroviral vectors enabled by an antisense cassette design preventing dsRNA formation during virus production

**DOI:** 10.1016/j.omta.2026.201719

**Published:** 2026-03-12

**Authors:** Romain Vuillefroy de Silly, Patrick Reichenbach, Melita Irving

**Affiliations:** 1 Ludwig Institute for Cancer Research, Department of Fundamental Oncology, University of Lausanne, Lausanne, Switzerland

**Keywords:** retrovirus, lentivirus, antisense, intron, polyadenylation, dsRNA, promoter, T cell, all-in-one

## Abstract

Retroviral vectors have demonstrated durable safety and therapeutic benefit in the context of cell and gene therapies, but achieving modular, independently controlled gene expression remains a major design challenge. Although antisense cassette architectures enable such regulation, yet they are limited by sharply reduced viral titers caused by double-stranded RNA (dsRNA) formation from convergent transcription during virus production. We developed an antisense cassette strategy that prevents dsRNA generation and preserves high titers. The design incorporates a sense-oriented intron containing an antisense polyadenylation signal into the transfer plasmid, enforcing premature termination of antisense transcription. This intron is subsequently spliced out of the packaged genomic RNA, thereby preventing inverted-promoter-driven dsRNA formation. The approach is compatible with both γ-retroviral and lentiviral vectors, supports diverse promoter combinations, and accommodates external introns as well as microRNA-based short hairpin RNAs for concurrent gene knockdown. As proof of concept, we engineered a dual-cassette vector containing an activation-inducible module paired with a constitutive transgene cassette, achieving robust modular expression alongside high titers. These findings establish a versatile platform enabling independent regulation of multiple genes in retroviral vectors and support the advancement of both basic research and therapeutic applications.

## Graphical abstract

**Figure:**
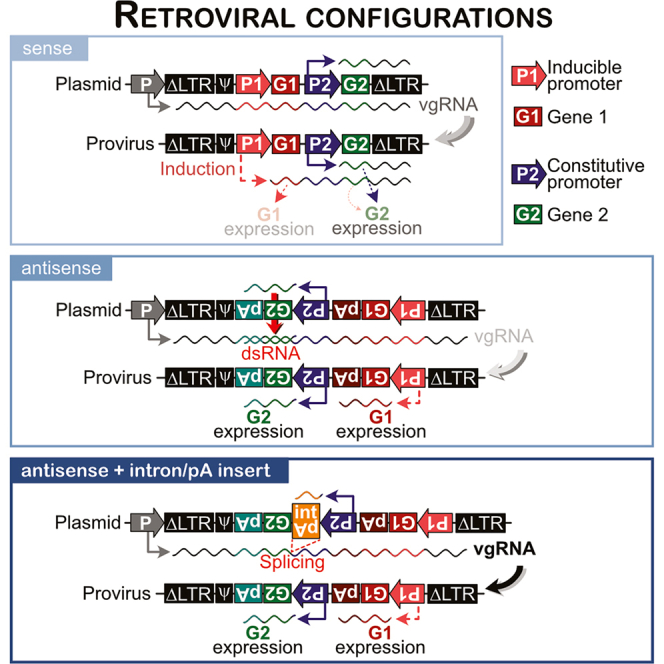

## Teaser

In this study, Vuillefroy de Silly and colleagues develop an antisense cassette for γ-retroviral and lentiviral vectors that prevents dsRNA formation during virus production, preserves high titers, and enables modular, independently regulated expression of multiple genes, providing a versatile platform for cell and gene therapy applications.

## Introduction

Retroviral vectors, including both γ-retroviral and lentiviral systems, are among the most widely used platforms for cell and gene therapies (CGTs) and have demonstrated an excellent safety profile in clinical settings.^1^^,^^2^^,^^3^^,^^4^ To expand the therapeutic potential of CGTs, there is a growing need for modular vector architectures that enable independent control of multiple genes through distinct promoters (e.g., constitutive, inducible, cell/tissue specific)^5^^,^^6^^,^^7^ without compromising transgene expression, viral titers, or product manufacturing. Such modular control is increasingly required across many CGT modalities, where precise and independent regulation of multiple genetic elements can enhance potency, safety, and therapeutic function. For instance, one may wish to constitutively express an antigen-targeting receptor under one Pol II promoter^5^^,^^7^ while inducibly driving secretion of a protein or/and concomitantly regulating endogenous gene expression via a Pol III-driven short RNA.^8^^,^^9^^,^^10^

We and others have previously shown that optimal, independently regulated Pol II transcriptional units in retroviral vectors can be achieved by inverting the expression cassette relative to the viral genomic RNA (vgRNA). This configuration prevents transcriptional interference^7^^,^^11^ between tandem promoters, avoids premature transcriptional termination that would otherwise truncate the viral genome during production, and preserves introns that would be lost in the sense orientation.^12^^,^^13^ Introns are valuable not only for enhancing transgene expression but also for embedding microRNA (miR)-based short hairpin RNAs (shRNAs) to couple endogenous gene knockdown (KD) with ectopic gene expression.^10^^,^^12^^,^^13^^,^^14^^,^^15^

A key limitation of antisense cassettes, however, is the formation of double-stranded RNA (dsRNA) during viral production, arising from convergent transcription initiated by the 5′ long terminal repeat (LTR) and the inverted promoter(s). This dsRNA leads to marked reductions in viral titers, largely due to RNA interference pathways.^7^^,^^13^^,^^16^^,^^17^ Although recent strategies have partially restored titers in lentiviral systems, analogous approaches suitable for γ-retroviral vectors have not been established. Moreover, approaches that rely on co-expression of third-party viral proteins, such as the Nodamura virus B2 protein (NoV B2) to suppress dsRNA responses, may not be compatible with clinical and good manufacturing practice (GMP) workflows.^7^

Here, we describe a versatile inverted-cassette design compatible with both γ-retroviral and lentiviral vectors. Our strategy incorporates a sense-oriented intron containing an antisense polyadenylation signal into the transfer plasmid, enabling premature termination of antisense transcripts during production, limiting dsRNA formation, and enhancing transgene expression. We demonstrate applicability across various antisense configuration designs for fine-tuned transgene expression levels, combinatory gene KD without loss of transgene expression, and combinations of constitutive and inducible promoters. Together, these advances provide a broadly adaptable platform for modular, independently regulated gene expression, offering considerable promise for next-generation CGT applications across multiple therapeutic modalities.

## Results

### Antisense cassette in the retroviral Q vector yields low viral titers

We recently developed an antisense lentiviral vector for independent expression of a chimeric antigen receptor (CAR) or T cell receptor (TCR) and an inducible transgene (e.g., a cytokine), along with a strategy for restoring viral titers.^7^ Briefly, during the production of lentivirus comprising an antisense cassette, both the 5′ LTR and the inverted promoter(s) are active, hence generating dsRNA by convergent transcription which (1) risks Dicer activation^18^ and (2) is limited to levels of single-stranded vgRNA available for packaging. To overcome these barriers to viral titers, we encoded the RNAi suppressor protein NoV B2 on the envelope protein vector to inhibit Dicer isoforms.^16^^,^^17^^,^^19^ In addition to favor transcription of vgRNA for packaging, we replaced the Rous sarcoma virus (RSV)-based promoter at the 5′ LTR with the cytomegalovirus (CMV) promoter and its enhancer which comprises 4 consensus NF-κB-binding motifs. We then included tumor necrosis factor (TNF) in the culture media during virus production which activates NF-κB in a dose-dependent manner, hence augmenting vgRNA levels.^7^^,^^20^

**Figure 1:**
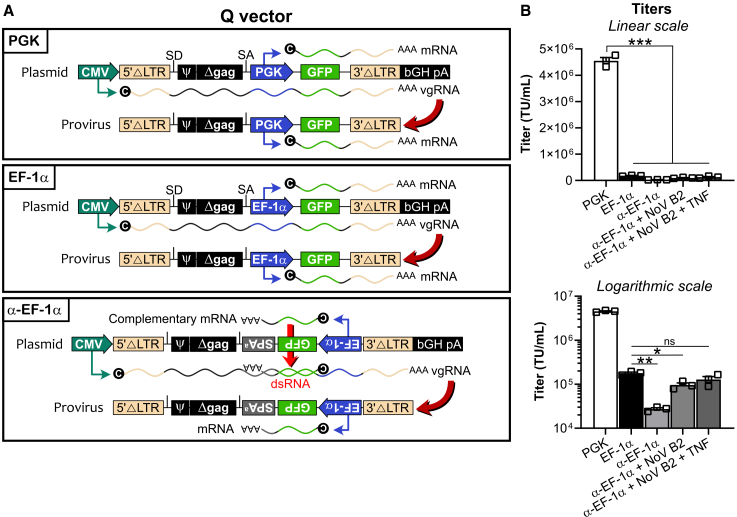
Design and titers of Q vectors comprising different expression cassettes (A) Scheme of the constructs in the Q vector backbone. Q vector was modified to include a bovine growth hormone polyadenylation (bGH pA) site after the 3′ long terminal repeat (LTR).^23^ Then, the green fluorescent protein (GFP)-expressing cassette included either (top), a human phosphoglycerate kinase (PGK) promoter (“PGK”), (middle), a human EF-1α promoter (“EF-1α”), or (bottom) an inverted cassette containing a human EF-1α promoter together with a synthetic polyadenylation site (SPA^a^) (“α-EF-1α”).^21^^,^^22^ The cassettes displayed each show (upper) the map of the plasmid during transfection and (lower) the map of the integrated provirus coming from the packaged viral genomic RNA (vgRNA). Transcripts arising from the CMV promoter (driving vgRNA) and from the internal promoter are displayed. The inverted cassette drives transcription of RNA complementary to vgRNA during virus production, leading to double-stranded RNA (dsRNA) generation. (B) Titers obtained from Q vector constructs. Bar graph shows the mean titers + SEM from three (symbols) independent experiments either in linear or logarithmic scale. In order to improve virus titer when using the antisense cassette, a plasmid allowing expression of Nodamura virus B2 protein (NoV B2) either co-transfected alone or in combination with tumor necrosis factor (TNF) addition (10 ng/mL) during virus production was assessed. ns, not significant, ∗*p* < 0.05, ∗∗*p* < 0.01, ∗∗∗*p* < 0.001 (Student’s paired *t* test). SD, splicing donor; SA, splicing acceptor; CMV, cytomegalovirus promoter; ΔLTR, deleted/mutated long terminal repeat; ψ, psi/packaging sequence; Δgag, deleted/mutated gag sequence.

To explore the use of an anti-sense cassette in the context of gamma-retrovirus, here, we began with the Q vector from Clontech/Takara Bio, the most commonly used self-inactivating (SIN) gamma-retroviral vector. In addition, we chose the elongation factor-1 alpha (EF-1α) as the antisense promoter given its strong transcriptional activity and the fact that it encompasses an intron. We inserted different cassettes in the Q vector to express green fluorescent protein (GFP) including (1) in sense orientation driven by the phosphoglycerate kinase (PGK) promoter (“PGK”; positive control), (2) in sense orientation driven by EF-1α (“EF-1α”), and (3), in antisense orientation driven by EF-1α (“α-EF-1α”) in conjunction with a synthetic polyadenylation site (“SPA^a^”) (Figure 1A).^21^^,^^22^ The rationale for adding an exogenous SPA to antisense expression cassettes is that, whereas sense retroviral vectors contain an active polyadenylation signal in the R region of the 3′ LTR, antisense retroviral vectors lack such a signal.

While the Q vector comprising the PGK promoter in sense orientation yielded high titers, switching to the EF-1α promoter resulted in ∼20 times lower levels (Figure 1B), possibly due to weak permissiveness of the CMV promoter in tandem transcription with EF-1α.^11^ The antisense cassette with EF-1α led to even lower titers, presumably as a consequence of dsRNA generation. Unlike as we had previously shown for antisense cassette lentiviral vectors, the use of NoV B2 (to limit RNAi) and TNF (to augment vgRNA levels)^16^^,^^17^ during antisense Q retrovirus production only modestly improved titers (Figure 1B).

### Q vector design to prevent dsRNA generation in the antisense configuration

**Figure 2:**
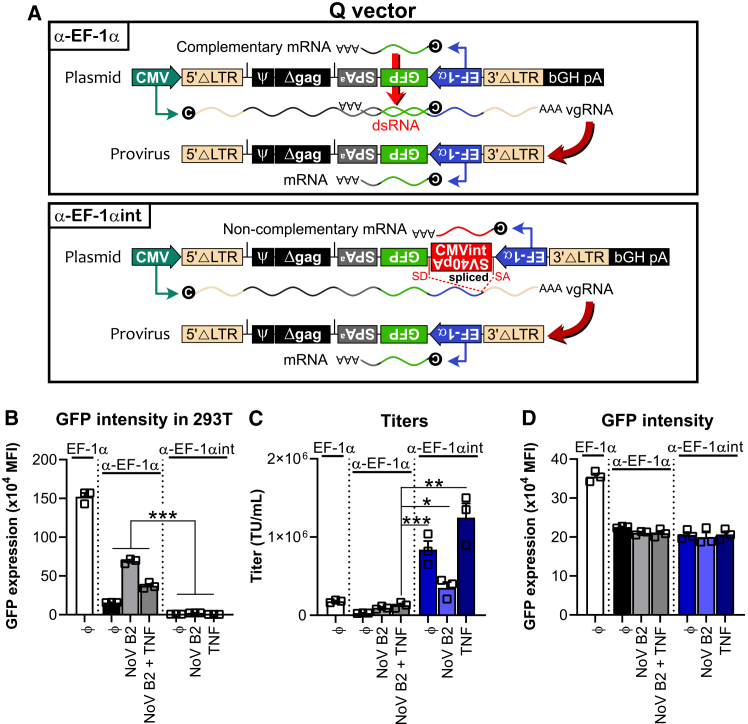
Insertion of an intron containing an inverted polyadenylation signal improves Q vector titers having an antisense cassette (A) Scheme of the antisense constructs in the Q vector backbone. (Top) The first design has an inverted cassette containing a human EF-1α promoter driving GFP expression together with a synthetic polyadenylation site (SPA^a^) (“α-EF-1α”). (Bottom) The second design (“α-EF-1αint”) includes a CMV intron A sequence (CMVint; directly preceding the inverted EF-1α promoter) containing an inverted late SV40 polyadenylation signal (SV40pA). (B) Analysis of GFP intensity in 293T cells during virus production. 293T cells were transfected with constructs containing sense EF-1α (“EF-1α”), antisense EF-1α (“α-EF-1α”) or antisense EF-1α containing the intron/α-SV40pA insert (“α-EF-1αint”), in the presence, or absence, of NoV B2 and/or TNF. Bar graph shows mean GFP fluorescence intensity in the GFP^+^ fraction + SEM from three independent experiments (symbols). ∗∗∗*p* < 0.001 (two-way ANOVA, Tukey post hoc test). (C) Analysis of viral titers. C1498 cells were transduced with viral supernatant from 293T cells transfected with constructs containing sense EF-1α (“EF-1α”), antisense EF-1α (“α-EF-1α”), or antisense EF-1α containing the intron/α-SV40pA insert (“α-EF-1αint”), in the presence, or absence, of NoV B2 and/or TNF. Bar graph shows mean titers + SEM from three independent experiments (symbols). ∗*p* < 0.05, ∗∗*p* < 0.01, ∗∗∗*p* < 0.001 (Student’s paired *t* test). (D) Analysis of GFP intensity upon C1498 transduction. C1498 cells were transduced with viral supernatant from 293T cells transfected with constructs containing sense EF-1α (“EF-1α”), antisense EF-1α (“α-EF-1α”), or antisense EF-1α containing the intron/α-SV40pA insert (“α-EF-1αint”), in the presence, or absence, of NoV B2 and/or TNF. Bar graph shows mean GFP fluorescence intensity in the GFP^+^ fraction + SEM from three independent experiments (symbols) where less than 20% cells were transduced.

Under the hypothesis that NoV B2 was insufficient in dampening the dsRNA response, we next sought to devise a strategy to prevent the formation of dsRNA in the first place during antisense cassette retrovirus production. To that end, directly preceding and downstream of the antisense EF-1α promoter, we inserted an intron (CMV intron A) in the sense orientation as well as a truncated antisense late SV40 polyadenylation (SV40pA) signal (Figure 2A, “α-EF-1αint”).^24^^,^^25^ As a consequence, while the intron and SV40pA signal insert would be spliced out during vgRNA production (i.e., generating a provirus cassette equivalent to the “α-EF-1α” construct), the EF-1α promoter would transcribe an unrelated RNA molecule (from the CMV intron) but would stop upon encountering SV40pA.

Next, in order to evaluate the ability of the intron/α-SV40pA insert to abrogate convergent transcription during retrovirus production, we analyzed GFP expression levels in 293T cells (Figure 2B). As expected, in comparison to the sense cassette (“EF-1α”) GFP expression was dramatically lowered for the antisense cassette (“α-EF-1α”) due to dsRNA generation and could be modestly restored in the presence of NoV B2. In contrast, for the antisense cassette including the intron/α-SV40pA insert (“α-EF-1αint”), GFP signal was abolished as intended, including in the presence of NoV B2 or TNF. Accordingly, the intron/α-SV40pA insert was able to boost viral titers to around 1 million transducing units (TU) per mL (Figure 2C). Adding TNF to the culture media further improved the titers, but we observed that NoV B2, employed to bind dsRNA and block Dicer-mediated cleavage,^19^^,^^26^ lowered viral titers. We speculate that NoV B2 may bind to dimerization domains and/or to secondary vgRNA structure and thereby prevent efficient packaging^27^^,^^28^ and titer restoration. Importantly, the fact that we observed similar GFP intensity for “α-EF-1αint” as “α-EF-1α” indicates that the intron/α-SV40pA insert was efficiently spliced out during virus production (Figure 2D).

### Development of an optimized SIN gamma-retroviral vector “RMS”

Given that “α-EF-1αint” Q vector-based gamma-retrovirus titers were lowered in the presence of NoV B2, and because the splicing donor (SD) and a splicing acceptor (SA) at the edges of the packaging/Δgag sequence could lead to aberrant splicing with the intron/α-SV40pA insert, we next sought to generate an optimized in-house SIN retroviral vector. Briefly, using the murine stem cell virus (MSCV)-based splice-gag vector (MSGV) (Figure S1A)^29^ as a backbone we (1) replaced the 5′-long terminal repeat (5′ LTR) U3 region by a RSV promoter, (2) abolished the SD and SA sites present at the edges of the psi/packaging and Δgag sequences, (3) removed the Δgag region, (4) inserted an optimized Woodchuck hepatitis virus post-transcriptional regulatory element (WPRE), (5) maximally truncated the 3′ LTR U3, and (6) added a bGH pA following the 3′ LTR.^23^^,^^30^^,^^31^^,^^32^

**Figure 3:**
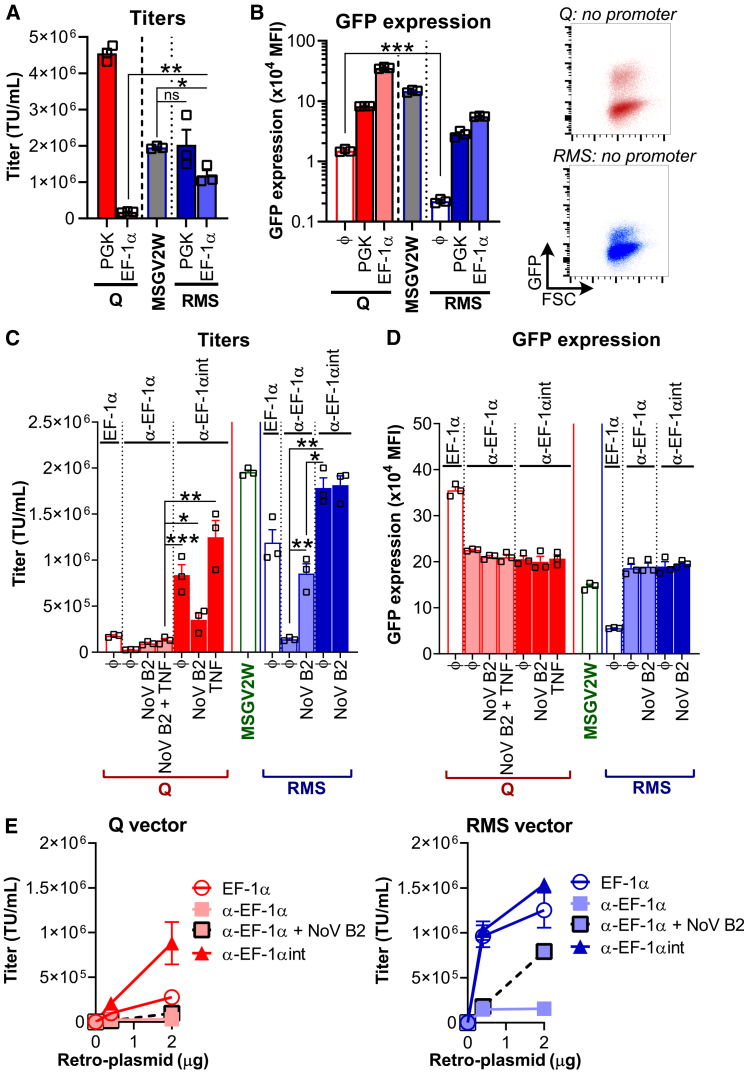
Creation of a SIN version from the MSGV gamma-retrovirus backbone (A) Comparison of viral titers. C1498 cells were transduced with viral supernatant from 293T cells transfected with various constructs bearing a sense orientation cassette. Bar graph shows mean titers + SEM from three independent experiments (symbols). ns, not significant, ∗*p* < 0.05, ∗∗*p* < 0.01 (Student’s paired *t* test). (B) Analysis of GFP intensity upon transduction. C1498 cells were transduced as in (A). Bar graph shows mean GFP fluorescence intensity in the GFP^+^ fraction + SEM from three independent experiments (symbols) where less than 20% cells were transduced. Representative dot plots display the GFP levels (as a function of cell size) observed when no internal promoter was added in the Q and RMS vectors. ∗∗∗*p* < 0.001 (Student’s paired *t* test). (C) Titer comparison between the Q and RMS vectors using EF-1α cassettes. C1498 cells were transduced with viral supernatants from 293T cells transfected with EF-1α, α-EF-1α or α-EF-1αint cassettes in the Q or RMS backbone, in the presence, or absence, of NoV B2 and/or TNF—in parallel, virus production from 293T cells transfected with the MSGV2W vector was also assessed. Bar graph shows the mean viral titer + SEM from three independent experiments (symbols). ∗*p* < 0.05, ∗∗*p* < 0.01, ∗∗∗*p* < 0.001 (Student’s paired *t* test). (D) GFP expression comparison between the Q and RMS vectors using antisense cassettes. Same experiments as in (C). Bar graph shows the mean GFP expression + SEM from three independent experiments (symbols) in the GFP^+^ population where less than 20% C1498 cells were transduced. (E) Virus production efficiency of Q and RMS retro-vectors. 0.4 or 2 μg of retro-vector was used for viral production (9.5 cm^2^ well). Graphs show the mean viral titer ± SEM from three independent experiments.

The RSV-driven MSGV-based SIN vector (“RMS” vector) with PGK as an internal promoter yielded similar strong titers as the parental MSGV2W vector (i.e., MSGV with WPRE) but lower as compared to the Q vector (Figure 3A). However, while titers plummeted for Q vector with EF-1α as an internal promoter, the RMS vector yielded approximatively 1 million transducing units per mL.

To assess self-inactivation level of the RMS vector, we examined GFP expression without inclusion of an internal promoter. We found GFP levels to be much lower for RMS than for the Q vector (Figure 3B), in line with U3 being more stringently truncated in RMS (i.e., RMS is more highly SIN than Q vector). The introduction of a PGK or EF-1α internal promoter in the Q vector led to higher GFP levels than for RMS, possibly resulting from the presence of a residual enhancer sequence and/or of a stronger polyadenylation signal. The EF-1α promoter sequence used in our study comprises an intron, and we questioned if it is more preferentially spliced out for either RMS or Q vector during virus production. Upon transduction, we observed both GFP^high^ and GFP^low^ populations (Figure S1B), presumably due to cells transduced with a full EF-1α promoter (unspliced) versus a short EF-1α promoter (spliced).^33^^,^^34^ Based on this assumption, the Q vector mostly generated viral particles comprising the unspliced EF-1α promoter and the RMS vector mostly the spliced version.

We next integrated an antisense cassette in the RMS vector (Figure 3C) and observed higher titers as compared to an equivalent Q vector. NovB2 augmented RMS viral titers but even higher titers were achieved for the antisense cassette RMS vector comprising the intron/α-SV40pA insert. Importantly, like for the Q vector shown previously, the insert was efficiently spliced out during virus production (Figure 3D). Finally, to determine the efficiency of virus production, we measured titers using 0.4 or 2 μg of vector (Figure 3E). While titers for the Q vector were linearly dependent on the quantity of vector used, reaching a maximum of 1 × 10^6^ TU/mL for 2 μg of αEF-1αint Q vector, this same titer was reached for αEF-1αint RMS at 0.4 μg vector, with titers of 1.5 × 10^6^ TU/mL achieved for 2 μg vector.

The high titers for “α-EF-1αint” RMS vectors can be explained by three important reasons. First, because the RMS vector was designed to prevent unwanted splicing out of the packaging sequence (which can occur for the Q vector), most RNA generated during virus production should be vgRNA suitable for packaging. Second, because splicing enhances nuclear export, the intron/α-SV40pA insert should promote packaging of viral particles. Finally, the intron/α-SV40pA insert abrogates convergent transcription, thereby favoring single-stranded vgRNA.

### Evaluation of antisense cassette RMS vector

**Figure 4:**
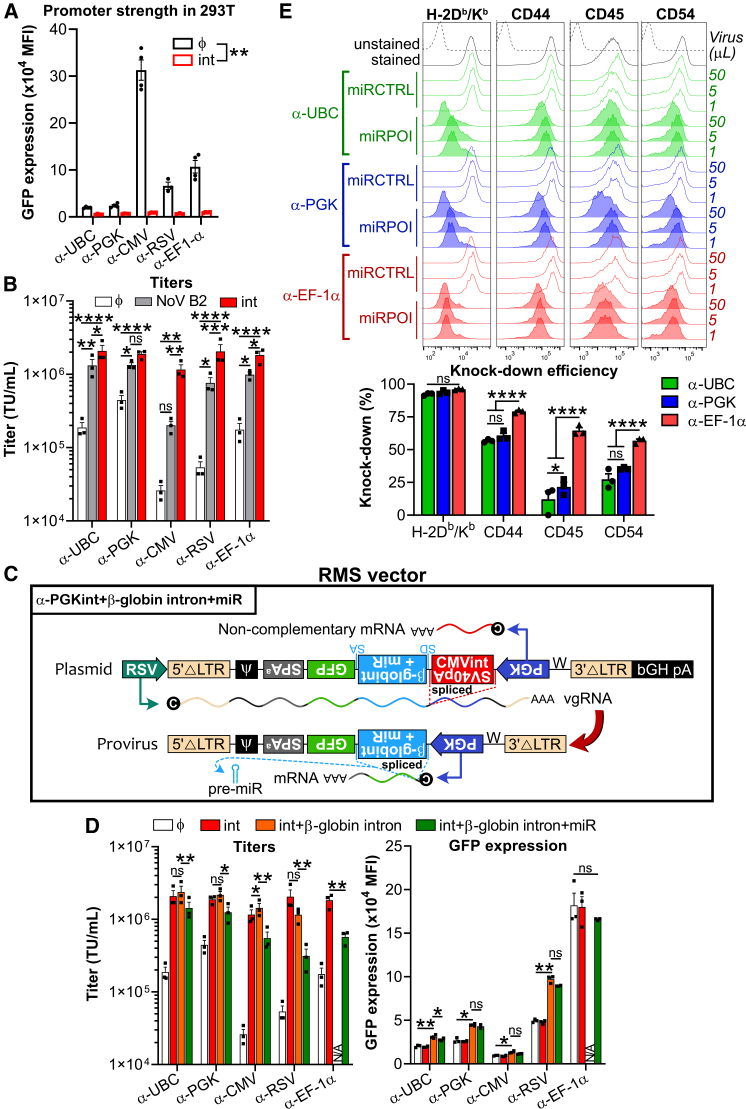
Antisense cassettes can accommodate introns and miR-based shRNAs with various promoters without a massive drop in viral titers (A) Analysis of GFP expression in 293T cells upon plasmid transfection. 293T cells were transfected with the RMS vector containing an antisense cassette (“ϕ”) that includes the GFP driven by UBC (α-UBC), PGK (α-PGK), CMV (α-CMV), RSV (α-RSV), or EF-1α (α-EF-1α) promoters. In parallel, 293T cells were transfected with plasmids comprising the same cassettes in which an intron containing an inverted polyadenylation site was added (“int”). Results show the mean GFP expression ±SEM from four independent experiments (symbols) 48 h after plasmid transfection. ∗∗*p* < 0.01 (Student’s paired *t* test; for each promoter, GFP expression comparison between the presence and the absence of the intron/α-SV40pA insert). (B) Viral titer determination. 293T cells were transfected with the RMS vector containing an antisense cassette (“ϕ”) that includes the GFP driven by UBC (α-UBC), PGK (α-PGK), CMV (α-CMV), RSV (α-RSV), and EF-1α (α-EF-1α) promoters. In parallel, these cells were transfected with an additional plasmid driving NoV B2 expression (“NoV B2”) or were transfected with plasmids in which an intron containing an inverted and premature pA site was added (“int”). 48 h after transfection, viral supernatants were used to determine viral titers upon transduction of C1498 cells. Results show the mean viral titer + SEM from three independent experiments (symbols) 72 h after transduction. ns, not significant, ∗*p* < 0.05, ∗∗*p* < 0.01, ∗∗∗*p* < 0.001, ∗∗∗∗*p* < 0.0001 (two-way ANOVA, Tukey post hoc test). (C) Representative scheme of an antisense cassette bearing a β-globin intron (“β-globint”) that includes a microRNA (miR)-based short hairpin RNA (shRNA; “miR”) in the RMS vector. During production of viruses the antisense cassette includes a premature pA signal preventing dsRNA generation. This premature pA is lost by splicing during viral genomic RNA production. Consequently, integrated viral genome leads to PGK-driven expression of GFP and to intronic pri-miR processing for target protein knockdown. W: Woodchuck hepatitis virus post-transcriptional regulatory element (WPRE). (D) Viral titer and GFP expression level analysis upon β-globin intron and miR-based shRNA inclusion. The set up was the same as in (B). In addition, an inverted β-globin intron without (“int+ β-globin intron”) or with a miR-based shRNA (“int+ β-globin intron+miR”) were inserted. Results show the mean viral titer (left graph) or mean GFP expression level (right graph, where less than 20% cells were transduced) + SEM from three independent experiments (symbols) 72 h after transduction. N/A, not assessed: β-globin intron was not added in the α-EF-1α condition, since the promoter already contains an intron. ns, not significant, ∗*p* < 0.05, ∗∗*p* < 0.01 (Student’s paired *t* test). (E) Knockdown efficiency depending on the promoter driving miR-based shRNA expression. β-globin intron and miR-based shRNA were inserted in antisense cassettes driven by UBC, PGK, and EF-1α promoters as in (D). Protein levels were analyzed by flow cytometry upon transduction of C1498 cells with miR-based shRNA sequences for various protein of interest (“miRPOI”): β2-microglobulin (a subunit needed for H-2D^b^/K^b^ expression), CD44, CD45, or CD54. Protein levels were compared to those obtained with a non-targeting miR-based shRNA control (“miRCTRL”). Representative flow cytometry histograms are shown depicting protein levels (from the reporter gene -expressing population) obtained upon infection with three doses of viral supernatants. Bar graph shows the mean knockdown efficiency + SEM from three independent experiments (symbols) 72 h after transduction where less than 20% cells were transduced. ns, not significant, ∗*p* < 0.05, ∗∗∗∗*p* < 0.0001 (two-way ANOVA, Tukey post hoc test).

Next, we sought to test the versatility of the antisense cassette RMS vector comprising the intron/α-SV40pA insert. We observed varying levels of transcriptional activity for the different promoters in 293T, with a hierarchy from highest to lowest of CMV > EF-1α > RSV > PGK > ubiquitin C (UBC) (Figure 4A) which largely corresponded to viral titer losses (Figure 4B). Viral titers could be augmented with NoV B2 for all promoters tested, but inclusion of the intron/α-SV40pA insert in the antisense cassette was consistently the most robust strategy for this while also efficiently blunting GFP expression during transfection (i.e., preventing convergent transcription).

An interesting feature of the antisense design is that introns can be maintained from transfection to transduction (sense orientation introns are spliced out) which can be leveraged to increase transgene expression or to include a miR-based shRNA without lowering transgene expression.^10^^,^^12^^,^^14^^,^^15^ Hence, we next included an antisense β-globin intron +/− miR-based shRNA (Figure 4C). With the β-globin intron, we observed higher GFP levels upon transduction and no negative impact on viral titers (Figure 4D). While slightly lowering viral titers, inclusion of miR-based shRNA enabled specific protein KD with efficiency that was promoter dependent and had low impact on the level of concomitant transgene expression upon transduction (Figure 4E).

### Building “all-in-one” dual antisense RMS vectors

Given the apparent versatility of the antisense RMS vector and our interest in co-engineering CAR- and TCR-T cells for treating solid tumors,^35^^,^^36^ we next sought to build and test an “all-in-one” dual antisense RMS vector in which a constitutive promoter drives expression of one protein (e.g., a CAR or TCR) and an inducible promoter drives the expression of a second one (e.g., an immunomodulatory cytokine).^37^

First, with the aim of maximizing transgene expression levels for the anti-sense RMS vector (Figures 2D and 3D), we tested another well-described synthetic polyadenylation signal^21^ which we sequence modified to disrupt a putative cryptic polyadenylation signal (“SPA^b^”) present in reverse orientation that could lower viral titers. Transduction of primary murine CD8^+^ OT-I T cells with RMS vectors comprising SPA^b^ versus SPA^a^ augmented transgene expression levels while not lowering viral titers (Figure S2A).

To benchmark the efficiency of our dual antisense RMS vector, we began by inserting a previously described all-in-one cassette into RMS.^5^^,^^7^ This all-in-one vector design, “mNFAT_EF-1α” (Figure S2B), includes a sense cassette comprising a synthetic inducible 6× murine NFAT promoter (mNFAT; 6 times repetition of the minimal mouse NFAT-binding motif together with a minimal Herpes simplex virus tyrosine kinase [HSV TK] promoter) which drives mCherry expression upon T cell activation.^38^^,^^39^ The mNFAT-mCherry is followed immediately by an EF-1α promoter driving GFP expression with the polyadenylation signal provided by the 3′ LTR.

In parallel, we adapted the RMS vector to include inverted expression of (1) inducible mNFAT-mCherry with the polyadenylation signal provided by a modified late SV40pA signal (targeted substitutions were introduced to maximize efficiency while precluding early SV40pA signal activity in reverse orientation)^24^^,^^25^ and (2) constitutive EF-1α-GFP and the polyadenylation signal SPA^b^. To this design (“α-mNFAT_α-EF-1α”; Figure S2B), we further included the intron/α-SV40pA insert (“α-mNFAT_α-EF-1αint”; Figure S2B) to abrogate dsRNA during virus production. Importantly, the SPA^b^ signal was not duplicated in the vector, and the intron/α-SV40pA insert is spliced out before retroviral reverse transcription, thereby minimizing the risk of viral recombination/template switching which frequently occurs during reverse transcription with homologous sequences.^40^ Finally, the 3 designs, “mNFAT_EF-1α,” “α-mNFAT_α-EF-1α,” and “α-mNFAT_α-EF-1αint,” were compared with those in the absence of the mNFAT cassette (“EF-1α,” “α-EF-1α,” and “α-EF-1αint”) to evaluate potential loss of transgene expression by transcriptional interference.^11^

**Figure 5:**
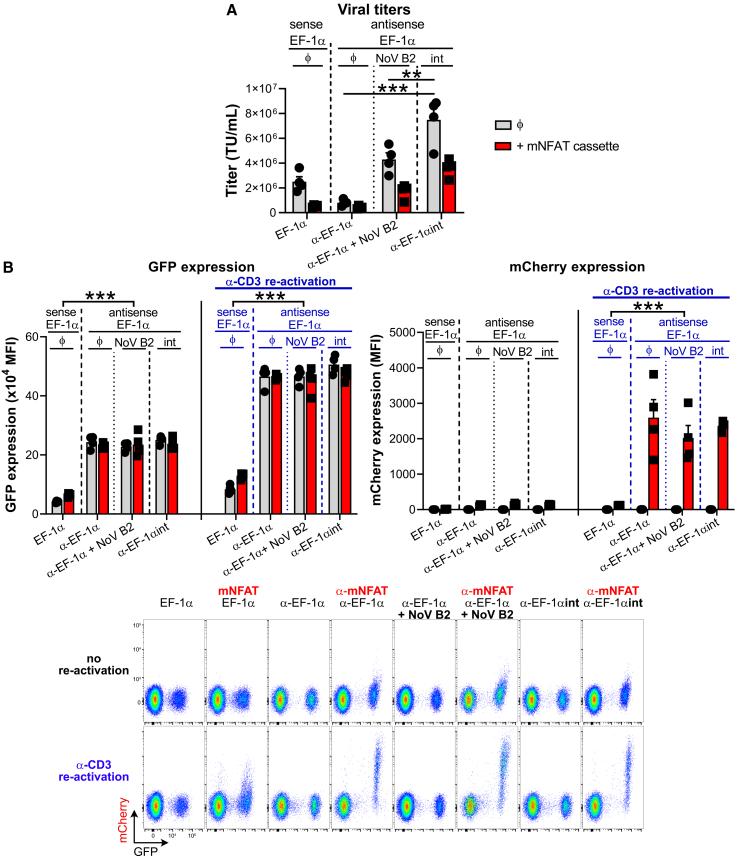
Design and testing of dual antisense retroviral vectors (A) Implementation of the antisense design together with the intron/α-SV40pA insert generates ‘all-in-one’ dual antisense retroviruses with high titers. Retroviruses were produced using RMS-based EF-1α, α-EF-1α (in the presence, or in the absence [“ϕ”], of NoV B2) or α-EF-1αint vectors in the presence (“+ mNFAT cassette”), or in the absence (“ϕ”), of a synthetic mouse NFAT promoter (mNFAT) (6 times repetition of the minimal mouse NFAT binding motif together with a minimal Herpes simplex virus tyrosine kinase promoter: promoter activity is TCR-signaling inducible) cassette. Viral titers were assessed upon transduction of pre-activated primary OT-I CD8^+^ T cells. Bar graph shows mean viral titer + SEM of four biological replicates (symbols) from two independent experiments. ∗∗*p* < 0.01, ∗∗∗*p*< 0.001 (Student’s paired *t* test). (B) Implementation of the antisense design provides a means for simultaneous, yet fully independent, transgene expression and leads to stronger expression from both, the constitutive and inducible, promoters. The setup was the same as in (A). Upon transduction, T cells were re-activated, or not, with plate-bound anti-CD3ε antibodies for 24 h. EF-1α -driven GFP and mNFAT-driven mCherry expressions were analyzed by flow cytometry in the GFP^+^ fraction. Bar graphs show mean GFP expression + SEM, or mean mCherry expression + SEM, of four biological replicates (symbols) from two independent experiments, where less than 20% cells were transduced. Representative dot plots obtained by flow cytometry are displayed. ∗∗∗*p* < 0.001 (Student’s paired *t* test; for matched conditions, comparisons between antisense and sense designs).

We observed that inclusion of 6× mNFAT-mCherry to both the sense and antisense vectors lowered viral titers (Figure 5A), which may be related to increased cargo size and/or secondary structures that could limit packaging efficiency. However, among the dual antisense designs, “α-mNFAT_α-EF-1αint” yielded the highest titers at approximately 4 × 10^6^ TU/mL. Upon transduction and re-activation (using anti-CD3 agonist antibodies) of primary murine CD8^+^ T cells to evaluate inducible and constitutive transgene expression (Figure 5B), we observed that the antisense designs led to highest GFP expression and that there was no promoter interference upon T cell activation from the mNFAT expression cassette. Notably, as has been described by others,^41^^,^^42^ T cell activation drove a 2-fold increase in constitutive EF-1α-driven GFP expression.

Upon T cell activation, low mCherry expression levels were observed for the sense “mNFAT_EF-1α” design, consistent with transcriptional interference, whereas robust mCherry induction was detected for the modular antisense designs. While some degree of mNFAT promoter “leakiness” was observed in the antisense configuration, this is likely attributable to basal activity of the promoter in non-activated T cells, which becomes apparent only in vector architectures that maximize transgene expression. In particular, the 6× murine NFAT promoter is a hybrid construct containing a minimal HSV TK promoter, which may confer basal transcription activity independent of activation signals. Importantly, the absence of an mCherry^+^ GFP^−^ population for “α-mNFAT_α-EF-1αint” indicates that most, if not all, of the intron/α-SV40pA sequence is efficiently spliced out during virus production.

Finally, given its wide use,^7^^,^^39^^,^^43^ we sought to evaluate the human synthetic 6× NFAT promoter (hNFAT: 6 times repetition of the minimal human NFAT-binding motif together with the minimal human IL-2 promoter) in our antisense RMS vector. Interestingly, while viral titers were similar (Figure S3A), mCherry induction was higher for hNFAT than for mNFAT (Figure S3B). However, the human 6× NFAT also displayed higher basal transcriptional activity in the absence of activation.

### Application of the antisense cassette to lentiviral vectors

**Figure 6:**
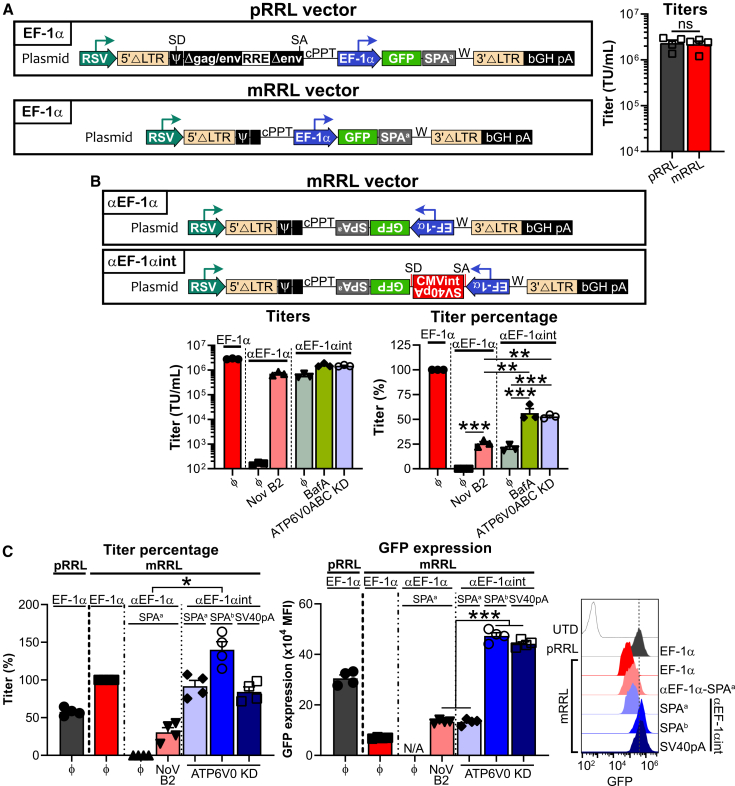
Application of the antisense strategy to a lentiviral vector (A) Schematic of pRRL features deleted and/or modified to generate mRRL (minimal pRRL) comprising an internal EF-1α-driven GFP expression cassette. mRRL was generated by deleting the splicing donor (SD) and splicing acceptor (SA), and by removing most of the gag and the whole env and the Rev response element (RRE). Titers were determined upon titration by flow cytometry with the Jurkat cell line. Bar graph shows the mean viral titer + SEM of four independent experiments (symbols). cPPT: central polypurine tract. ns, not significant (Student’s paired *t* test). (B) Schematic of antisense cassettes used in the mRRL vector comprising an inverted EF-1α-driven GFP expression cassette. 293T cells were transfected with mRRL vector carrying EF-1α (“ϕ”), αEF-1α (“ϕ”) or αEF-1αint cassettes. Restoration of titers was assessed in the α-EF-1α condition by co-transfection with a NoV B2-expressing plasmid. For the αEF-1αint cassette, titer improvement was further assessed either upon acute treatment of 293T cells with Bafilomycin A1 (20 nM; “BafA”) for 3 h 16–18 h post-transfection followed by medium change, or upon co-transfection with a mix of miR-based shRNA plasmids targeting ATP6V0A2, ATP6V0B, and ATP6V0C (“ATP6V0ABC KD”). Titers were determined upon titration by flow cytometry with the Jurkat cell line. Bar graphs show the mean viral titer + SEM as transducing units per mL or percentage, of three independent experiments (symbols). ∗∗*p* < 0.01, ∗∗∗*p*< 0.001 (Student’s paired *t* test). (C) Polyadenylation signal refinement improves transgene expression while preserving viral titers. 293T cells were transfected with the pRRL vector carrying an EF-1α cassette, or with mRRL vectors carrying EF-1α (“ϕ”), αEF-1α (“ϕ”), or αEF-1αint cassettes. Impact of SPA^a^, SPA^b^, or SV40pA was assessed with the αEF-1αint design. Restoration of titers was carried out in the αEF-1α condition by co-transfection with a NoV B2-expressing plasmid. For the αEF-1αint cassettes, titers were further improved via co-transfection with a mix of miR-based shRNA plasmids targeting ATP6V0A2, ATP6V0B, and ATP6V0C (“ATP6V0 KD”). Titers were determined upon titration by flow cytometry with the Jurkat cell line. Bar graphs show the mean viral titer percentage or mean GFP expression + SEM of four independent experiments (symbols). One representative experiment displaying GFP expression as detected by flow cytometry is shown. ∗*p* < 0.05, ∗∗∗*p*< 0.001 (Student’s paired *t* test; for titers, comparisons between antisense constructs and antisense constructs containing an intron/α-SV40pA insert).

Finally, we sought to test our antisense cassette in lentiviral vectors given their ability to infect a broad range of human cell types, both dividing and non-dividing, and their extensive use in clinical CGT. To accommodate our intron splicing-based strategy, we began with critical modifications to the lentiviral pRRL vector,^44^^,^^45^^,^^46^ including (1) abrogation of the SD and SA sites surrounding the packaging and Δenv regions; (2) removal of most of Δgag; (3) removal of Δenv^47^; and, most importantly, (4) removal of the Rev response element (RRE) which upon binding in *trans* has been described to prevent splicing and facilitate export and packaging of unspliced RNA transcripts (Figure 6A, left).^48^^,^^49^ Despite that these modifications to generate vector mRRL (minimal pRRL) accounted for more than 1,000 bp deletions, there was no lowering of viral titers (Figure 6A, right).

Subsequently, we introduced the antisense cassette into mRRL (Figure 6B, left). We observed that unlike for RMS gamma-retrovirus, the intron/α-SV40pA insert could not fully restore mRRL lentiviral titers, achieving similar yields as using NoV B2 (Figure 6B). While the gamma-retrovirus in this study was pseudotyped with an ecotropic envelope (i.e., murine tropism), the lentivirus was pseudotyped with the vesicular stomatitis virus G glycoprotein (VSV-G), which has the ability to enter a broad range of cell types, including human, and to mediate high titer gene transfer. Hence, we hypothesized that during lentivirus production in 293T cells, the release of viral progeny (no longer comprising the intron/α-SV40pA insert) and their retro-transduction of the transfected 293T cells^50^ may be generating dsRNA and a consequent drop in titer.

To mitigate progeny retro-transduction during lentivirus production in 293T cells, we evaluated two strategies. First, because VSV-G-mediated fusion for cytoplasmic release is pH dependent,^51^ we attempted to limit endosome acidification by acute treatment of transfected 293T cells with Bafilomycin A1 for 3 h before the medium change that is routinely carried out 16–18 h post-transfection. In parallel, because Bafilomycin A1, a specific vacuolar H^+^-ATPase (V-ATPase) inhibitor binding to the V0 sector, is not GMP compliant, we also concomitantly knocked-down subunits A2, B, and C of the V0 sector (via co-transfection with miR-based shRNA plasmids) to abolish V-ATPase activity (Figure 6B). Interestingly, despite not fully restoring titers, Bafilomycin A1 use and V0 subunits KD led to further (and equivalent) improvement in viral titers for antisense cassette lentivirus comprising the intron/α-SV40pA insert.

Lastly, we evaluated transgene expression for the antisense cassette mRRL lentiviral vector, comparing polyadenylation signals SPA^a^, SPA^b^, and SV40pA as done previously for the dual antisense RMS gamma-retroviral vector (Figure 5; Figure S2). Interestingly, we observed higher GFP expression levels in the context of SPA^b^ and SV40pA than in the pRRL vector, while there was no loss in viral titers with these changes (Figure 6C). Taken together, these data show that our antisense cassette comprising the intron/α-SV40pA insert is fully applicable to lentiviral vectors.

## Discussion

Achieving modular, multi-gene control within a single retroviral vector is a key requirement for next-generation CGTs. Our findings show that an antisense configuration provides this capability by ensuring defined transcript composition, boosting expression, and enabling the integration of introns and miR-based shRNA modules within one vector. This design enables precise control over the final mRNA composition, eliminating residual vector-derived sequences that could negatively influence expression. We observed marked increases in transgene expression, particularly when a strong polyadenylation signal was incorporated: an approach that is not compatible with sense-oriented vector designs, as the introduction of an exogenous polyadenylation signal in this context would interfere with viral genome transcription and result in inefficient virion production lacking an intact 3′ LTR. Previous studies have shown that intron inclusion can be necessary for optimal therapeutic expression,^13^ and here, the antisense orientation allowed stable intron retention in the integrated provirus. This not only enhanced expression but also enabled efficient incorporation of miR-based shRNA modules for concurrent gene KD and transgene delivery. Moreover, promoter choice within the antisense configuration provided a means to fine-tune both transgene expression and KD efficacy.

The ability to combine multiple transcriptional units within a single vector greatly expands retroviral modularity. We previously showed that multi-gene expression in T cells using lentiviral vectors required antisense cassettes to achieve independent, non-interfering control of each gene.^7^ Sense-oriented cassettes suffered from transcriptional interference, while bidirectional promoters exhibited leakiness. Here, we extended this approach to develop an all-in-one antisense retroviral vector that coordinates a constitutive transgene with an inducible one, using both murine and human 6×NFAT promoters.

A major drawback of antisense cassettes is the substantial loss in viral titers caused by Dicer-dependent responses to dsRNA generated during virus production. We previously addressed this issue in lentiviral vectors by enhancing vgRNA transcription using a CMV promoter and TNF in the culture media and by suppressing RNAi with the NoV B2 protein.^7^ In the present study, we attempted to apply these strategies to the commercially available Q vector, a SIN γ-retroviral backbone, but found that neither NoVB2 nor TNF effectively restored titers. This prompted us to develop an alternative strategy to prevent convergent transcription and thus dsRNA formation at its source. We show that introducing a sense-oriented intron (CMV intron A) together with an antisense SV40 polyadenylation signal (intron/α-SV40pA insert) downstream of the antisense EF-1α promoter induces premature termination of antisense transcription and enables intron splicing from the viral genome, collectively preventing dsRNA generation.

We further demonstrated that this intron/α-SV40pA strategy effectively restored titers in an optimized γ-retroviral SIN vector (RMS, derived from pMSGV) and a modified lentiviral vector (mRRL, derived from pRRL). These streamlined vectors lack major splice donor/acceptor sites flanking the ψ/env region and, in the case of mRRL, the RRE. Although both vectors remained partially responsive to NoV B2, titer recovery was consistently superior with the intron/α-SV40pA strategy. Importantly, this approach proved broadly compatible with multiple promoters of varying strengths, including UBC, PGK, RSV, EF-1α, and CMV.

A remaining limitation is that the efficiency of the intron/α-SV40pA design depends partly on the pseudotype and the retro-transduction susceptibility of producer cells such as 293T. While titers were fully restored with an ecotropic envelope, VSV-G pseudotyping yielded substantial but incomplete recovery. We found that titers could be further enhanced by inhibiting VSV-G-mediated fusion through transient treatment with the V-ATPase inhibitor bafilomycin A1 or by knocking down V-ATPase subunits (ATP6V0A2, ATP6V0B, and ATP6V0C), thereby limiting endosomal acidification.^51^ KD of V-ATPase subunits represents a more clinically translatable approach, as it is more compatible with GMP-compliant settings and does not require substantial modification of the existing manufacturing workflow. In practice, KD plasmids could be co-transfected alongside the transfer, packaging, and envelope plasmids during virus production. Moreover, the corresponding KD-encoding sequences could be incorporated directly into the transfer vector, thereby preserving the overall manufacturing workflow. An alternative strategy would involve removing specific envelope receptors from producer cells to prevent retro-transduction during production.

Together, these findings provide an important step forward in the design of all-in-one retroviral vectors that offer enhanced modularity while maintaining strong viral titers. High-titer single-vector systems have major implications for reducing manufacturing costs, simplifying GMP workflows, and improving product homogeneity by eliminating the need for co-transductions. Furthermore, the ability to combine ectopic gene overexpression with targeted downregulation of endogenous genes within a single vector opens new opportunities to improve the potency, persistence, and safety of adoptive cellular therapies.

## Materials and methods

### Mice

OT-I (Charles River) mice, which carry a TCR transgene specific for ovalbumin, were housed at the University of Lausanne (UNIL, Epalinges, Switzerland) animal facility. All experiments were conducted in accordance and approval of the Service of Consumer and Veterinary Affairs (SCAV) of the Canton of Vaud.

### Cell culture

The 293T, C1498, and Jurkat cell lines (ATCC) were grown in DMEM medium containing 4.5 g/L glucose, sodium pyruvate, and glutamax (Gibco), supplemented with 10% heat-inactivated fetal bovine serum (PAN Biotech), 10 mM HEPES, 50 U/mL penicillin, and 50 μg/mL streptomycin (Gibco).

### Plasmids

The Q vector from Takara/Clontech was modified to contain a bovine growth hormone polyadenylation (bGH pA) signal after the 3′ LTR. pCMV-NovB2 plasmid, allowing production of the Nodamura virus B2 (NoV B2) protein, includes a CMV promoter, and an intron and a polyadenylation signal from the rabbit β-globin. The gamma-retroviral ecotropic packaging plasmid pCL-Eco was a gift from Inder Verma (Addgene plasmid # 12371; http://n2t.net/addgene:12371; RRID: Addgene_12371),^52^ the lentiviral packaging plasmid pCMVR8.74 (Addgene plasmid #22036; http://n2t.net/addgene:22036; RRID:Addgene_22036) and the VSV-G envelope-expressing plasmid pMD2.G (Addgene plasmid # 12259; http://n2t.net/addgene:12259; RRID:Addgene_12259) were gifts from Didier Trono. MSGV2W plasmid was derived from the MSGV retroviral vector.^29^ The pRRL vector that we used is coming from the pRRLSIN.cPPT.PGK-GFP.WPRE vector, which was a gift from Didier Trono (Addgene plasmid # 12252; http://n2t.net/addgene:12252; RRID:Addgene_12252). The full list of constructs used in the study is provided in Table 1, and a sequence list of new modules and constructs is summarized in Table S1.

**Table 1:** Construct list

*N°*	Construct name	*N°*	Construct name
1	Q - no promoter GFP	32	RMS - αRSVint GFP
2	Q - PGK GFP	33	RMS - αRSVint b-globin GFP
3	Q - EF-1α GFP	34	RMS - αCMVint b-globin miRCTRL GFP
4	Q - αEF-1α GFP	35	RMS - EF-1α GFP
5	MSGV2W GFP	36	RMS - αEF-1α GFP
6	RMS - no promoter GFP	37	RMS - αEF-1α GFP SPA^b^
7	RMS - UBC GFP	38	RMS - αEF-1αint GFP
8	RMS - αUBC GFP	39	RMS - αEF-1αint GFP SPA^b^
9	RMS - αUBCint GFP	40	RMS - αEF-1αint b-globin GFP
10	RMS - αUBCint b-globin GFP	41	RMS - αEF-1αint b-globin miRCTRL GFP
11	RMS - αUBCint b-globin miRCTRL GFP	42	RMS - αEF-1αint b-globin miRb2m GFP
12	RMS - αUBCint b-globin miRb2m GFP	43	RMS - αEF-1αint b-globin miRCD44 GFP
13	RMS - αUBCint b-globin miRCD44 GFP	44	RMS - αEF-1αint b-globin miRCD45 GFP
14	RMS - αUBCint b-globin miRCD45 GFP	45	RMS - αEF-1αint b-globin miRCD54 GFP
15	RMS - αUBCint b-globin miRCD54 GFP	46	RMS - mNFAT mCherry_EF-1α GFP
16	RMS - PGK GFP	47	RMS - α-mNFAT mCherry_α-EF-1α GFP
17	RMS - αPGK GFP	48	RMS - α-mNFAT mCherry_α-EF-1αint GFP
18	RMS - αPGKint GFP	49	RMS - α-hNFAT mCherry_α-EF-1αint GFP
19	RMS - αPGKint b-globin GFP	50	pRRL - EF-1α GFP
20	RMS - αPGKint b-globin miRCTRL GFP	51	mRRL - EF-1α GFP
21	RMS - αPGKint b-globin miRb2m GFP	52	mRRL - αEF-1α GFP
22	RMS - αPGKint b-globin miRCD44 GFP	53	mRRL - αEF-1αint GFP
23	RMS - αPGKint b-globin miRCD45 GFP	54	mRRL - αEF-1αint GFP SPA^b^
24	RMS - αPGKint b-globin miRCD54 GFP	55	mRRL - αEF-1αint GFP SV40pA
25	RMS - CMV GFP	56	pCMV - NoV B2
26	RMS - αCMV GFP	57	pAO2 - U6L miRATP6V0A2
27	RMS - αCMVint GFP	58	pAO2 - U6L miRATP6V0B
28	RMS - αCMVint b-globin GFP	59	pAO2 - U6L miRATP6V0C
29	RMS - αCMVint b-globin miRCTRL GFP	60	pCL-Eco
30	RMS - RSV GFP	61	pCMVR8.74
31	RMS - αRSV GFP	62	pMD2.G

#### Construction of a new self-inactivated gamma-retroviral vector: RMS

An SIN vector was further derived from the MSGV2W plasmid, called RMS (RSV-driven MSGV-based SIN vector). Briefly, (1) the U3 sequence of the 5′ LTR was replaced with the Rous sarcoma virus (RSV) promoter, (2) the splicing donor located downstream of the primer-binding site was destroyed, (3) downstream of the psi/packaging sequence the whole truncated/mutated gag sequence was removed (including the splicing acceptor site), (4) the human phosphoglycerate kinase (PGK) promoter or elongation factor-1 alpha (EF-1α) was added, (5) most of the U3 region from the 3′ LTR was deleted, and (6) the bGH pA site was added directly after the U5 region of the 3′ LTR.

#### Construction of a new minimal pRRL vector: mRRL

From the pRRL vector, we derived a minimal pRRL (mRRL) version that lacks most of the envelope and gag sequence and the RRE and in which the original WPRE sequence is swapped with the optimized WPRE version.

#### Antisense cassettes

In order to improve titers when using the antisense configuration, a cassette composed of a modified CMV intron A that includes an inverted version of the late SV40pA signal (and shortened in order to avoid premature transcription termination given by the early SV40pA in the sense orientation),^24^^,^^25^ was included (intron/α-SV40pA). Whenever included, it was inserted 10–30 nucleotides after promoter-specific transcription start. The antisense sequence of the late and shortened α-SV40pA was

ATCATAATCAGCCATACCACATTTGTAGAGGTTTTACTTGCTTTAAAAAACCTCCCACACCTCCCCCTGAACCTGAAACATAAAATGAATGCAATTGTTGTTGTTAACTTGTTTATTGCAGCTTATAATGGTTACAAATAAAGCAATAGCATCACAAATTTCACA.

Other antisense polyadenylation signal sequences used were

• synthetic polyadenylation signal (SPA^a^): CACACAAAAAACCAACACACATCCATCTTCGATGGATAGCGATTTTATT
• synthetic polyadenylation signal (SPA^b^): CTAGACACACAAAAAACCAACACACAGATCTAATGAAAACAAAGATCTTTTATTGCTAG
• late SV40pA signal with substitutions (SV40pA): GATCATAATCAGCCATACCACATTTGTAGAGGTTTTACTTGCTTTAAAAAACCTCCCACACCTCCCCCTGAACCTGAAACATAAAATGAATGCAATTGTTGTTGTTAACTTGTTTATTGCAGCTTATAATGGTTACAAACAAAGCAATAGCATCACAAATTTCACAAACAAAGCATTTTTTTCACTGCATTCTAGTTGTGGTTTGTCCAAACTCATCAATGTATCTTATCATGTCTG

Whenever we added a rabbit β-globin intron, the sequence was slightly modified to prevent undesired splicing events. Addition of a miR-30a-based shRNA was carried out by inserting it in the β-globin intron or in the EF-1α intron. In order to clone the selected shRNA sequences (from http://splashrna.mskcc.org)^53^ into the miR-30a backbone, hybridized primers encompassing the passenger, the loop, and the guide sequences were ligated into the PaqCI pre-digested vector.^10^ Whenever we assessed KD efficacy, we used the Thy-1.1 reporter gene instead of GFP.

#### ATP6V0 KD plasmids

The miR-based shRNAs against ATP6V0A2, B and C were expressed in the a MSGV2W cloning plasmid backbone in which the entire viral sequence from the start of the 5′ LTR to the end of the 3′ LTR was removed. The plasmid contained the human U6-driven (including the leader sequence)^54^ miR-30a backbone (including PaqCI restriction sites as previously described) and a minimal Simian virus 40 (SV40) origin of replication.^55^ The sequences of the hybridized primers to obtain the final miR-based shRNAs were as follows.

• miRATP6V0A2-F: AGCGCTCCCCTCATTCATGAATATAATAGTGAAGCCACAGATGTATTATATTCATGAATGAGGGGAT
• miRATP6V0A2-R: GGCAATCCCCTCATTCATGAATATAATACATCTGTGGCTTCACTATTATATTCATGAATGAGGGGAG
• miRATP6V0B-F: AGCGCTCCTAGTGTTTGTGAAATAAATAGTGAAGCCACAGATGTATTTATTTCACAAACACTAGGAA
• miRATP6V0B-R: GGCATTCCTAGTGTTTGTGAAATAAATACATCTGTGGCTTCACTATTTATTTCACAAACACTAGGAG
• miRATP6V0C-F: AGCGACAGCCACAGAATATTATGTAATAGTGAAGCCACAGATGTATTACATAATATTCTGTGGCTGG
• miRATP6V0C-R: GGCACCAGCCACAGAATATTATGTAATACATCTGTGGCTTCACTATTACATAATATTCTGTGGCTGT

### Virus production and titration

293T were transfected using the calcium phosphate technique^56^ with a 1:1 (2 μg of each for a 9.5 cm^2^ well) retroviral transfer vector to packaging plasmid (pCL-Eco) ratio for gamma-retroviruses or a 3:2:1 lentiviral transfer vector to packaging plasmid (pCMVR8.74) to envelope plasmid (pMD2.G) ratio for lentiviruses. Medium was replaced one day later and supernatants were harvested two days post-transfection to assess titers. In some conditions, pCMV-NovB2 (1:0.5 transfer vector to NoV B2 plasmid ratio) or an equimolar mixture of plasmids encoding miR-based shRNAs against ATP6V0A2, B and C (1:1 transfer vector to miR pool plasmids ratio) were co-transfected. TNF (Peprotech; 10 ng/mL) was added during the whole process of transfection and viral production when combined to NoV B2. In order to inhibit V-ATPase during lentiviral production, one day after transfection Bafilomycin A1 (Santa Cruz, sc-201550; 20 nM) was added to the supernatant of 293T cells for 3 h before medium change. The next day, lentiviral supernatant was collected for titration. Retroviral or lentiviral content was titrated (based on GFP positivity by flow cytometry) by serial dilution of viral supernatants with the C1498 or Jurkat cell line, respectively, in the presence of 10 μg/mL protamine sulfate (Sigma-Aldrich, P4020). Viral titers were calculated under conditions yielding a transduction efficiency below 25% (percent GFP^+^ cells), corresponding to a single integration event per cell. Titers (TU/mL) were calculated using the following formula: Viral titer = Transduction efficiency × seeded cell number ÷ supernatant volume.

### Splenocyte isolation, CD8^+^ selection, transduction, and re-activation

Splenocytes were extracted from OT-I mice upon spleen isolation, dissociation (smashing spleens through a 70 μm cell strainer), and red blood cell lysis (using hypotonic solution containing 0.15 M ammonium chloride, 10 mM potassium bicarbonate, and 0.1 mM EDTA). CD8^+^ T cells were purified by negative selection using the Biolegend MojoSort mouse CD8^+^ T cell isolation kit (480008; >90% purity) and were primed by culture on plates pre-coated with anti-CD3ε antibodies (5 μg/mL; clone 145-2C11, BioLegend) in the presence of anti-CD28 antibodies (1 μg/mL; clone 37.51, BioLegend) at 37°C with 5% CO2. After two days, cells were transduced in the presence of 10 μg/cm^2^ of retronectin (Takara) following manufacturer’s instructions and recombinant murine IL-2 was added (200 IU/mL; PeproTech). The next day, cells were transferred to another plate and diluted in the presence of exogenous IL-2. Seven days post-priming, cells were activated for 24 h with anti-CD3ε-coated (1 μg/mL) plates.

### Flow cytometry

The buffer used for flow cytometry studies was composed of PBS, 0.5% BSA, 0.1% NaN_3_, and the staining procedure included the use of a live/dead fixable cell stain kit (Molecular Probes) to analyze viable cells. For surface marker expression, cells were stained with anti- H-2K^b^/H-2D^b^-PE (114607), CD44-APC (103011), CD45RB-APC (103319), CD54-APC (116119), and CD90.1/Thy-1.1-BV421 (202529) antibodies from BioLegend. Samples were run using a CytoFlex (Beckman Coulter) flow cytometer. Analyses were carried out on singlets using FlowJo software, and marker expression was calculated from the median fluorescence intensity (MFI). KD efficacy was calculated relative to the MFI obtained with the miRCTRL condition as follows: KD (%) = 100 - (MFI miR of interest × 100/MFI miRCTRL). In order to assess miR efficacy upon one viral copy integration per cell, a titration with miR-expressing viruses was carried out and MFI values were analyzed in conditions for which less than 25% C1498 cells were transduced.

### Statistical analysis

Statistical significance was evaluated using the two-tailed paired Student’s *t* test for comparison of two groups or a two-way ANOVA with a Tukey post hoc test for comparison of multiple groups using the GraphPad Software (Prism).

## Data and code availability

The data presented in this article are available upon request to the corresponding author.

## Acknowledgments

We thank the Flow Cytometry Facility of the University of Lausanne. We sincerely thank and acknowledge the financial support for this work from 10.13039/100009729Ludwig Cancer Research, the 10.13039/501100006390University of Lausanne, the 10.13039/501100001711Swiss National Science Foundation (SNSF# 310030_204326), the 10.13039/501100017035ISREC Foundation, and the Fondazione Teofilo Rossi di Montelera e di Premuda.

## Author contributions

R.V.d.S., conceptualization, data curation, formal analysis, supervision, investigation, visualization, methodology, writing, and project administration. P.R., resources. M.I., resources, supervision, funding acquisition, writing, and project administration.

## Declaration of interests

A provisional patent regarding the retroviral vectors and associated methodologies for increasing virus titers as described in this manuscript has been filed (2025 European Patent Application no. 25190914.9 “Retroviral vectors and methods for a higher titer production”) with R.V.d.S. and M.I. as co-inventors.

## Supplemental information

Document S1. Figures S1–S3

Table S1. Construct/module sequences used in the study

Document S2. Article plus supplemental information
