# High-titer modular retroviral vectors enabled by an antisense cassette design preventing dsRNA formation during virus production

**DOI:** 10.1016/j.omta.2026.201797

**Published:** 2026-06-30

**Authors:** Romain Vuillefroy de Silly, Patrick Reichenbach, Melita Irving

## Main text

(Molecular Therapy: Advances *34*, 1–15; June 2026)

In the originally published version of this article, Figure 6A incorrectly showed the presence of a synthetic polyadenylation signal (SPAa) in the pRRL and mRRL sense-oriented expression cassettes. This feature is not present in the actual constructs. Figure 6A has been corrected accordingly by removing the SPAa element from the schematic.

**Figure:**
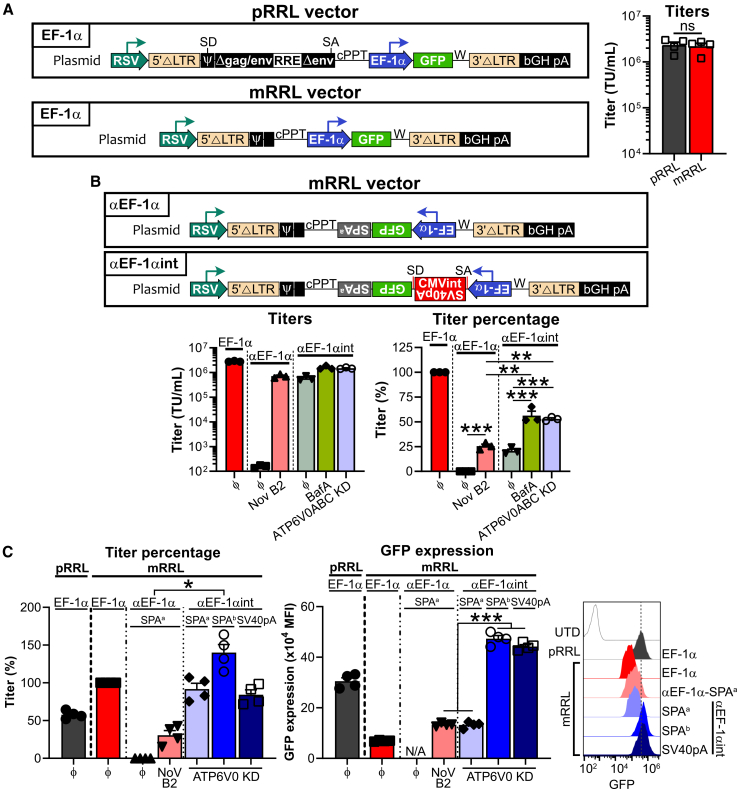
Figure 6. Application of the antisense strategy to a lentiviral vector (corrected)

**Figure:**
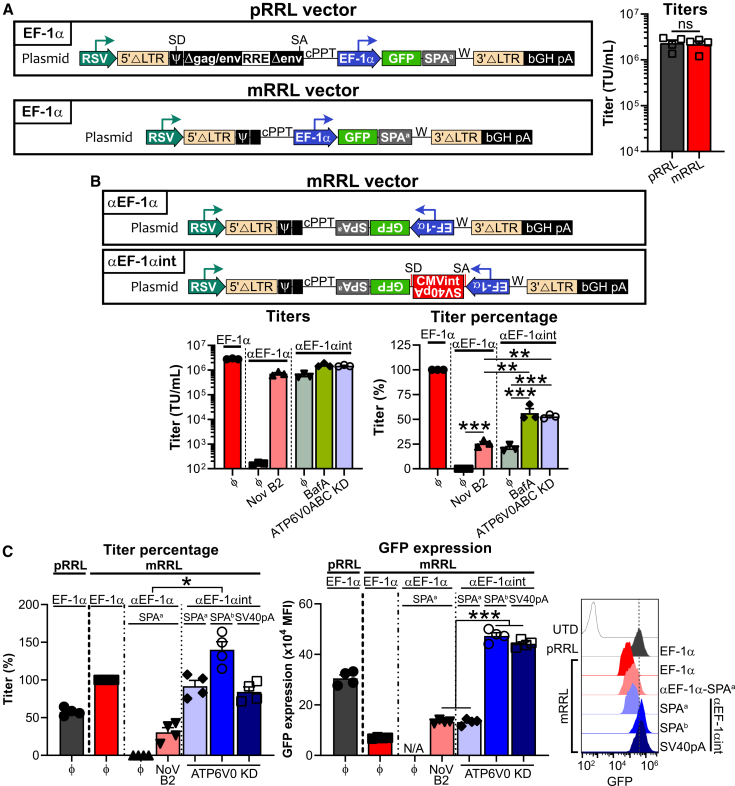
Figure 6. Application of the antisense strategy to a lentiviral vector (original)

