# Supplementary material for: High-titer modular retroviral vectors enabled by an antisense cassette design preventing dsRNA formation during virus production
Source: Mol Ther Adv. 2026 Mar 12;34(2):201719. doi: 10.1016/j.omta.2026.201719 (PMC13148933; doi:10.1016/j.omta.2026.201719)
Supplement: Document S1. Figures S1–S3 [file mmc1.pdf]

OMTA, Volume *34*

Supplemental information

High-titer modular retroviral vectors enabled by an antisense cassette design preventing dsRNA formation during virus production

Romain Vuillefroy de Silly, Patrick Reichenbach, and Melita Irving

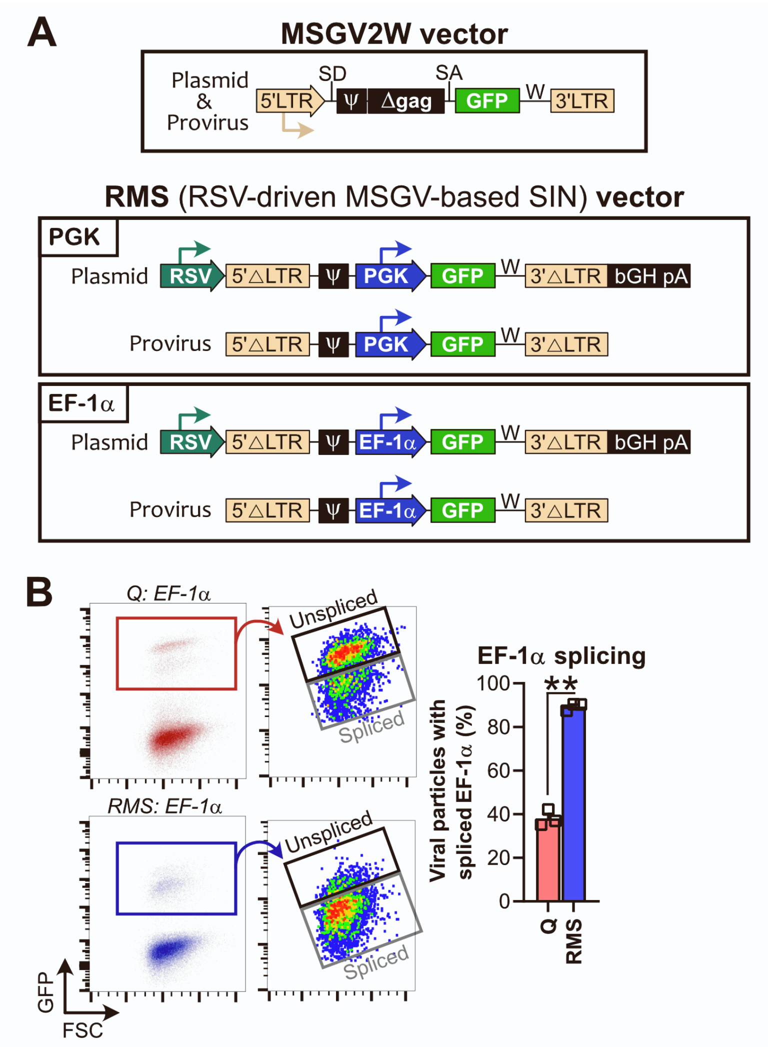

Figure S1. Development of RMS, a SIN vector derived from the MSGV gamma-retrovirus backbone. (**A**) Schematic of MSGV-based vectors. MSGV2W is derived from MSGV by adding a WPRE (W) sequence before the 3'LTR. RSV-driven MSGV-based SIN (RMS) vector was created by adding an RSV promoter in place of U3 at the 5'LTR, removing splicing donor (SD) and acceptor (SA) sites, removing the gag sequence, adding an internal promoter and a WPRE sequence before the 3'LTR, deleting most of the U3 from the 3'LTR, and adding a bGH pA sequence downstream of the 3'LTR. (**B**) EF-1α splicing efficiency depending on vector backbone. C1498 cells were transduced with viral supernatant from 293T cells transfected with sense EF-1α cassette (for which the intron can be spliced out during virus production) in the Q or RMS backbone. Representative dot plots on the left display the GFP levels (as a function of cell size) observed upon transduction. Representative dot plots in the middle display the gating used to estimate EF-1α splicing efficiency based on GFP^{high} and GFP^{low} C1498 populations in the GFP^{+} fraction. Bar graph shows mean percent splicing out of EF-1α intron from the GFP^{+} fraction + SEM from three independent experiments (symbols) where less than 20% cells were transduced. **: p<0.01 (Student's paired *t*-test).

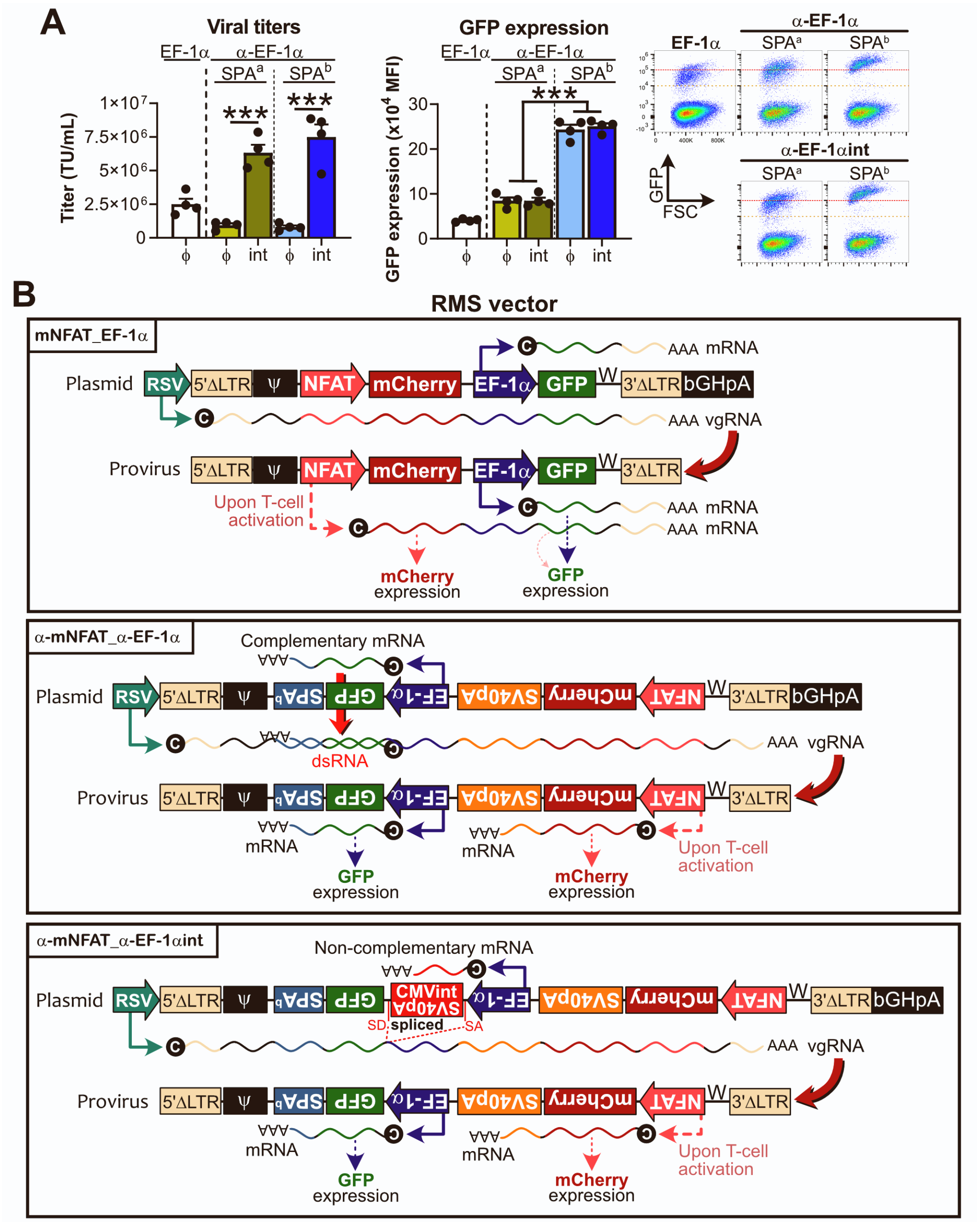

Figure S2. 'All-in-one' dual antisense RMS vector design. (**A**) Polyadenylation signal refinement improves transgene expression while preserving viral titers. Pre-activated OT-I CD8$^+$ T cells were transduced with retroviruses encoding GFP with EF-1α, α-EF-1α or α-EF-1αint cassettes in the RMS backbone. Antisense transcriptional termination was obtained with SPA^a or SPA^b sequences. Bar graphs show mean viral titers + SEM, or GFP expression MFI (in the GFP$^+$ fraction) + SEM from four biological replicates (symbols) out of two independent experiments. Representative dot plots of the GFP levels (as a function of cell size) are displayed (where less than 20% cells were transduced). ***: p<0.001 (Student's paired *t*-test). (**B**) Scheme of the RMS-based 'all-in-one' dual antisense vectors. "mNFAT_EF-1α" vector consists of a synthetic mouse NFAT promoter (mNFAT) (6 times repetition of the minimal mouse NFAT binding motif together with a minimal Herpes simplex virus tyrosine kinase promoter: promoter activity is TCR-signaling inducible), mCherry, the EF-1α promoter and GFP. "α-mNFAT_ α-EF-1α" vector is composed of antisense sequences of: mNFAT, mCherry, SV40pA, EF-1α, GFP and SPA^b. "α-mNFAT_ α-EF-1αint" vector is equivalent to the "α-mNFAT_ α-EF-1α" vector, but possesses an additional intron/α-SV40pA module to prevent EF-1α-driven complementary RNA generation during viral production.

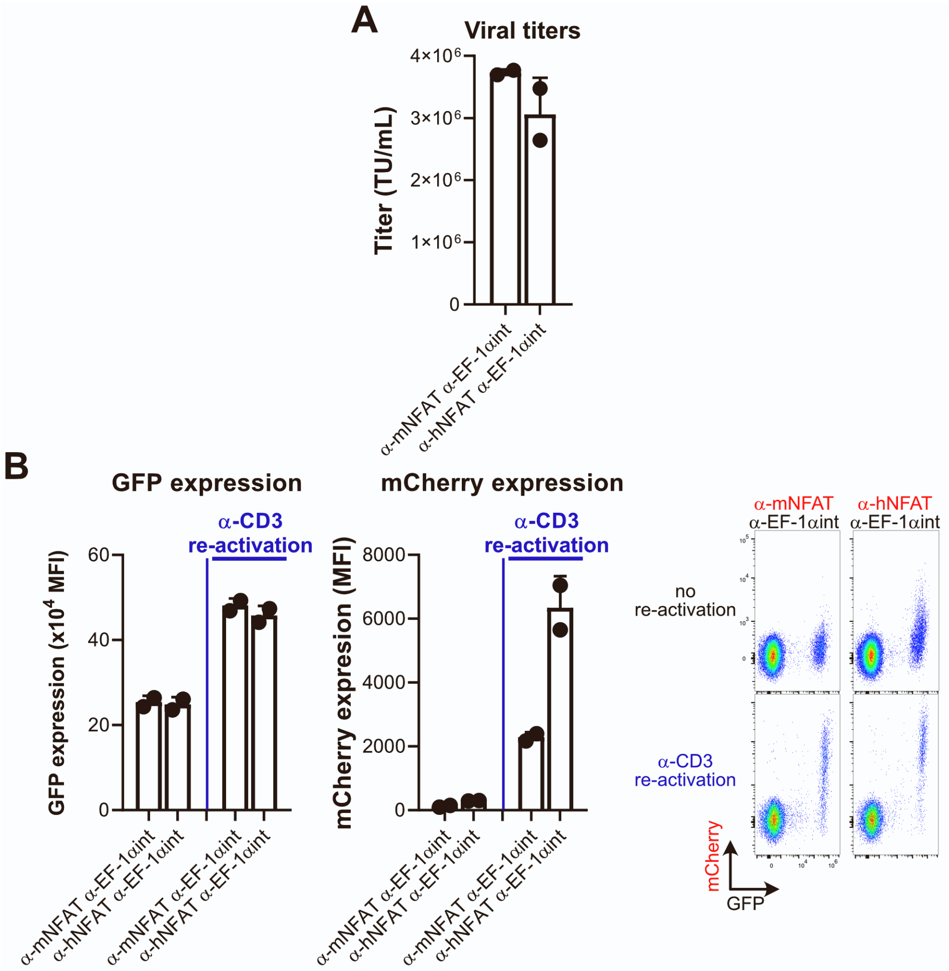

Figure S3. Human versus mouse synthetic NFAT promoters in dual antisense retroviral vectors. (**A**) Similar viral titers for antisense RMS vectors comprising human versus mouse synthetic NFAT promoters. Retroviruses were produced using RMS-based α-EF-1αint vectors in the presence of a mouse (α-mNFAT) or a human (α-hNFAT) NFAT cassette. Viral titers were assessed upon transduction of pre-activated primary OT-I $CD8^+$ T cells. Bar graph shows mean viral titer + SEM of two biological replicates (symbols) from two independent experiments. (**B**) Human synthetic NFAT promoter leads to higher mCherry expression than for mouse NFAT. The setup was the same as in (A). Upon transduction, T cells were re-activated, or not, with plate-bound anti-CD3ε antibodies for 24 hours. EF-1α -driven GFP and NFAT-driven mCherry expressions were analyzed by flow cytometry in the GFP^+ fraction. Bar graphs show mean GFP expression + SEM, or mean mCherry expression + SEM, of two biological replicates (symbols) from two independent experiments, where less than 20% cells were transduced. Representative dot plots obtained by flow cytometry are displayed.

Table S1. Construct/ module sequences used in the study.
